# Risk of Birth Defects From Vitamin A “Acne Supplements” Sold Online

**DOI:** 10.5826/dpc.1103a75

**Published:** 2021-05-20

**Authors:** Dina H. Zamil, Emily K. Burns, Ariadna Perez-Sanchez, Milbrey A. Parke, Rajani Katta

**Affiliations:** 1 Baylor College of Medicine, Houston, Texas, USA; 2 Department of Internal Medicine, University of Texas Health Science Center at San Antonio, San Antonio, Texas, USA; 3 McGovern Medical School at University of Texas Health Science Center at Houston, Houston, Texas, USA

**Keywords:** vitamin A, acne supplements, teratogenicity, pregnancy, labeling

## Abstract

**Background:**

Dietary supplements are popular among US consumers and claim to address a variety of conditions, including acne. Acne supplements containing vitamin A are of particular interest, due to the potentially teratogenic effects of vitamin A doses over 10,000 IU.

**Objective:**

This study examined dosage, pregnancy risks, and labeling of vitamin A-containing acne supplements available online.

**Methods:**

An Internet search of acne supplements sold online was conducted between March and May 2020. Supplement labels and websites were analyzed for vitamin A content and pregnancy warnings, and then divided into categories based on dosage and teratogenic risk.

**Results:**

A total of 49 acne supplements was found, and of these 26 (53%) contain vitamin A. Three supplements are likely teratogenic, 4 contain vitamin A doses exceeding the daily level of intake that meets the nutritional needs of most people, and 15 have an unknown teratogenic risk. Among the 6 supplements with over 10,000 IU vitamin A, 2 have no pregnancy warning at all, including the supplement with the highest vitamin A dose found in this study.

**Conclusions:**

Dietary supplements are not subject to the same stringent regulations as drugs, and as such, consumers may be unaware of pregnancy risks. Furthermore, FDA requirements on labeling of vitamin A supplements may lead to consumer confusion regarding dosage. As such, we encourage stricter labeling requirements for vitamin A-containing supplements, including pregnancy warnings for high-dose supplements and clearer dosage labeling.

## Introduction

In the United States, over half of adults and one-third of children use dietary supplements [1,2]. Although dietary supplements are not evaluated with the same rigor as medications by the US Food and Drug Administration (FDA), they remain popular [3,4]. In fact, one survey of national public opinion found that many users believed supplements to be so effective that even if clinical data suggested inefficacy, they would continue to consume them [3]. As such, the dietary supplement market is booming, with over 90,000 supplements on the market in the US in 2014 [5]. Indeed, dietary supplements claiming to address a variety of diseases can be purchased easily, in retail stores or online [6].

Many dietary supplements found online claim to target acne. In a survey among acne patients visiting a dermatology clinic, many respondents believed that supplements could help with acne, particularly vitamin A supplements [7]. Dietary supplements containing vitamin A commonly report their dosages in either international units (IUs) or retinol activity equivalents (RAE).

Over-the-counter (OTC) acne supplements that contain high-dose vitamin A could be unsafe, especially during pregnancy. Supplemental doses of vitamin A greater than 10,000 IU (3,000 mcg RAE) have been found to be particularly dangerous during the first trimester of pregnancy: approximately 1 in 57 babies born to women consuming more than 10,000 IU of supplemental preformed vitamin A daily had attributable birth defects [8].

The objective of this study was to investigate vitamin A-containing acne supplements currently available online and to identify supplements with potentially teratogenic doses. This study also sought to examine issues with labeling and regulation of vitamin A supplements.

## Methods

An online search of acne supplements sold online was conducted between March 2020 and May 2020. Supplements including the terms “whitehead,” “blackhead,” and “acne” were independently searched on Amazon, Google, Instagram, and Twitter. The products found on Google, Instagram, and Twitter were linked to their manufacturer websites. Nutrition label information for each supplement was analyzed using descriptions and photos of product labels on manufacturer and third-party websites. All products that included the terms “whitehead,” “blackhead,” or “acne” in the photograph of the product label or in the online description were included in the study. Supplements were examined for vitamin A content, including daily dose and forms of vitamin A. These details were used to categorize acne supplements according to dose size. The teratogenic risk of each product was calculated by converting dosages to IUs or RAE, when possible.

Thresholds for dose categorization included tolerable upper intake levels (ULs) and recommended daily allowances (RDAs) as well as the estimated teratogenic threshold for vitamin A [8,9]. The threshold for teratogenicity was set at 10,000 IU preformed vitamin A or 3,000 mcg RAE equivalent, based on a study of vitamin A intake and birth defects among babies born to over 22,000 pregnant women [8]. Supplements exceeding this dose are considered high risk. Supplements with high doses were divided by vitamin A form (preformed or precursor). Supplements with unknown risk were classified into 5 subcategories: (a) information in IUs provided, but teratogenicity risk unknown because the form of vitamin A was not specified; (b) information in IUs provided, but not enough information available to calculate mcg RAE; (c) confusing labeling (difficult to understand the relative proportions of vitamin A forms or amount of vitamin A in dose); (d) labeling with discrepancies; and (e) unspecified dose.

Labels and websites for supplements with suspected teratogenic risk were thoroughly searched for pregnancy warnings. Products with confusing labels, discrepancies in nutrition information, unspecified vitamin A form or dose, or too little information to determine teratogenicity were also identified.

## Results

A total of 49 unique acne supplements were identified, and 26 (53.1%) included some form of vitamin A. The forms of vitamin A contained in the acne supplements included vitamin A acetate, retinol acetate, retinyl acetate, beta-carotene, vitamin A palmitate, retinol palmitate, retinyl palmitate, and natural mixed carotenoids. The dose of vitamin A provided by these supplements ranged from 120 mcg RAE to 21,000 mcg RAE. The vitamin A-containing acne supplements were divided into 4 main categories, based on their potential teratogenicity (Table 1). Overall, 3 supplements exceeded the threshold for teratogenicity (category 1), and 4 supplements with high doses of vitamin A precursors, not known if teratogenic but of concern given the lack of research, were placed in category 2. The study found 11 supplements with unknown risk and 12 supplements with low doses of vitamin A that are unlikely to be teratogenic and pose low risk.

Among the 6 supplements with over 10,000 IU vitamin A (3 in category 1 and 3 in category 3), 2 have no pregnancy warning at all, including the supplement with the highest vitamin A dose in this study. Another 2 of these supplements contain warnings on both the product bottle and website. However, these merely urge users to consult a physician prior to consumption if pregnant. For one of these supplements, a third-party website (but not the product bottle or product website) displays a California Proposition 65 warning, disclosing that the product contains chemicals known to cause reproductive harm or birth defects. Proposition 65 requires businesses to warn Californian consumers about teratogenic products [10]. Finally, 2 supplements have warnings advising consumers not to take the supplement if pregnant, one on both the bottle and website, the other only in website material.

## Discussion

This study found 10 acne supplements sold online that contain relatively high doses of vitamin A, 3 of which are likely teratogenic. Physicians need to educate patients that purchasing “acne supplements” online may pose risks. Dietary supplements do not require FDA approval before going to market [11]. In fact, a manufacturer may market a supplement without providing any proof of safety or efficacy [12]. Additionally, while prescription medications are required to carry pregnancy warning categories, no such requirement is in place for supplements, even for those that carry a risk of teratogenicity. Finally, current FDA supplement labeling regulations for vitamin A are confusing, making it difficult for consumers to evaluate teratogenic risk.

### Risks of Teratogenicity

Vitamin A may be found in the form of either preformed vitamin A or vitamin A precursors [9]. Preformed vitamin A includes retinol and retinyl esters. Vitamin A precursors include provitamin A carotenoids, such as beta-carotene [9].

Given the lack of deficiency of vitamin A within the United States, the World Health Organization does not recommend routine vitamin A supplementation in general [13]. Guidance suggests that vitamin A supplementation should be limited to a dose under 5,000 IU per day, given the risks of excess vitamin A supplementation [14].

Current knowledge of teratogenicity risk posed by excess vitamin A is based on studies evaluating consumption of only preformed vitamin A, measured in IUs [8]. The safety of high-dose beta-carotene, a vitamin A precursor, is not known. Regarding the teratogenicity of beta-carotene, according to an expert consensus [15], the toxic effects typically associated with high-dose vitamin A are not associated with high-dose beta-carotene; nonetheless, the public is advised to remain cautious of potential adverse effects of beta-carotene. In fact, the US Food and Nutrition Board urges against beta-carotene supplementation as its ULs have not been established [9]. The FDA has concluded that beta-carotene found in supplements has equivalent retinol activity to preformed retinol. One major concern, therefore, regarding supplemental beta-carotene is whether its teratogenicity risk is also similar to retinol. This concern has not been resolved, as human studies performed to date have only evaluated retinol, not supplemental beta-carotene [8,9,16]. However, of note, one recent animal study of embryonic development indeed found that nano-encapsulated beta-carotene induced craniofacial and eye birth defects, and beta-carotene in bulk formulation resulted in developmental delays [17].

Drugs exhibiting evidence of teratogenicity are labeled pregnancy category D or X. Category X is reserved for drugs with pregnancy risks that outweigh its benefits [18]. Historically, several vitamin A derivative medications were placed in category X, including etretinate and isotretinoin. Etretinate was even removed from the US market for safety reasons [14, 19].

In marked contrast, teratogenic supplements, such as the 3 identified in this study, do not have to display pregnancy category warnings to comply with labeling legislation [8,20]. While some potentially dangerous acne supplements sold online were found to have some form of warning, these were often inadequate, simply recommending consultation with a physician before use. It is quite easy for consumers to overlook these warnings in the “fine print,” especially if the warning is not on the product bottle. For supplements with an extremely high vitamin A dose, a category X warning on websites, bottles, and capsules would be more appropriate and easier for consumers to find.

### Concerns About Current Labeling

#### Labeling Discrepancies

Several supplements have issues with labeling. For instance, one supplement has no dosage of vitamin A listed. Others have labeling discrepancies. Two products provide conflicting versions of the Supplement Facts label. For one of these supplements, one version of the label displays a correct percent of the daily value (% DV), and the other displays an incorrect % DV [21, 22]. Another product listed on 2 different websites has different Supplement Facts labels on each website. One of these supplement labels provides a dose of retinyl palmitate on a third-party website that is less than half of the dose provided on the manufacturer’s website.

#### FDA regulations on the labeling of vitamin A supplements may lead to confusion

Previously, vitamin A levels were reported in IUs [9]. To facilitate comparison of the biological activity of different forms of vitamin A, the Food and Nutrition Board created a new measure called RAE [9]. This measure replaced IUs as of January 2020 for companies with $10 million or more in annual sales [9].

To convert IUs to mcg RAE, knowledge of the vitamin A source is necessary [9]. Converting from IUs to mcg RAE and vice versa is important because the estimated threshold for teratogenicity is reported in IUs [8]. In this study, 2 supplements provided vitamin A dosage in mcg RAE, but did not provide enough information to convert to IUs, making it difficult to evaluate the teratogenic risk. Three other supplements provided doses in IUs, but not enough details to convert to mcg RAE.

We did not rely on % DV when calculating vitamin A doses. While % DV should refer to mcg RAE for vitamin A, assuming the manufacturer is in compliance with regulations, the word “RAE” is not required on the label [21, 22]. These regulations complicate labels and may lead to confusion on the part of consumers and healthcare professionals attempting to determine the amount of vitamin A in a supplement. We identified 4 supplements available for sale online with confusing labeling with regards to vitamin A.

#### Incorrect Labeling

Three supplements with incorrect labeling were identified. One supplement indicates the amount of retinyl acetate in addition to the vitamin A dose in mcg RAE. However, by our calculations, the amount of retinyl acetate indicated on the label does not equal the calculated vitamin A dose in mcg RAE. Another supplement label provides a vitamin A dose in mcg RAE that is consistent with its displayed % DV [21, 22]. The vitamin A dose provided in IUs on the label, however, is not equivalent to the dose provided in mcg RAE [9]. Finally, one supplement indicates the amount of beta-carotene it contains in IUs, but it also lists vitamin A as a separate ingredient, without specifying its amount or form.

## Conclusions

The nutrition labels of acne supplements sold online can have discrepancies, may be confusing, or may be incorrect with regards to vitamin A content. Given the known teratogenic effects of high-dose vitamin A, consumers of child-bearing potential should remain cautious about the consumption of acne supplements containing vitamin A. Some OTC acne supplements containing teratogenic doses of vitamin A fail to display adequate warnings. The FDA labeling regulations for vitamin A supplements should require more stringent requirements for pregnancy warnings as well as clearer dosage labeling. After all, public opinion surveys have shown support for increased FDA surveillance and regulation of dietary supplements [3].

## Figures and Tables

**Table 1:** Classification of 26 Vitamin A-containing Acne Supplements Sold Online

Dosage Criteria	No. of Supplements^b^
**1. High doses of preformed vitamin A.**^a^ ≥ 3,000 mcg RAE or more, which is the UL for preformed vitamin A in adults ≥ 19 years as well as a potentially teratogenic dose [1,2]	3
**2. High doses of vitamin A precursors.** a >900 mcg RAE, the RDA of vitamin A (both preformed and precursors) for males ≥ 14 years [2]	4
**3. Unknown risk**	
Information in mcg RAE provided, but not enough information available to calculate IUs	2
Information in IUs provided or can be calculated, but teratogenicity risk unknown because form of vitamin A or relative proportions of vitamin A forms not specified. >10,000 IU vitamin A	3
Information in IUs provided, but not enough information to convert to mcg RAE	3
Confusing labeling	4
Labeling with discrepancies	3
Dosage not specified	1
**4. Low doses of vitamin A**	
For females. <700 mcg RAE (both preformed and precursors), which is the RDA for females ≥14 years [2]	6
For males. <900 mcg RAE (both preformed and precursors), which is the RDA for males ≥14 years [2]	9

IUs = international units; RAE = retinol activity equivalents; RDA = recommended daily allowance; UL = tolerable upper intake level.

a Measured by mcg RAE or IUs.

b Some supplements were placed into more than one category
